# The Ichikado CT score as a prognostic tool for coronavirus disease 2019 pneumonia: a retrospective cohort study

**DOI:** 10.1186/s40560-021-00566-4

**Published:** 2021-08-21

**Authors:** Alan Araiza, Melanie Duran, Cesar Patiño, Paul E. Marik, Joseph Varon

**Affiliations:** 1 United Memorial Medical Center, Houston, TX USA; 2 Universidad Autónoma de Baja California, Tijuana, México; 3 Universidad Xochicalco, Ensenada, México; 4 Benemérita Universidad Autónoma de Puebla, Puebla, México; 5 University of Texas Health Science Center at Houston, Houston, TX USA

**Keywords:** Coronavirus disease 2019, COVID-19, CT score, Ichikado CT score, COVID-19 prognostic factor, Tomography

## Abstract

**Background:**

The relationship between computed tomography (CT) and prognosis of patients with COVID-19 pneumonia remains unclear. We hypothesized that the Ichikado CT score, obtained in the first 24 h of hospital admission, is an independent predictor for all-cause mortality during hospitalization in patients with COVID-19 pneumonia.

**Methods:**

Single-center retrospective cohort study of patients with confirmed COVID-19 pneumonia admitted at our institution between March 20th, 2020 and October 31st, 2020. Patients were enrolled if, within 24 h of admission, a chest CT scan, an arterial blood gas, a complete blood count, and a basic metabolic panel were performed. Two independent radiologists, who were blinded to clinical data, retrospectively evaluated the chest CT scans following a previously described qualitative and quantitative CT scoring system. The primary outcome was all-cause in-hospital mortality or survival to hospital discharge. Secondary outcomes were new requirements for invasive mechanical ventilation and hospital length of stay. Cox regression models were used to test the association between potential independent predictors and all-cause mortality.

**Results:**

Two hundred thirty-five patients, 197 survivors and 38 nonsurvivors, were studied. The median Ichikado CT score for nonsurvivors was significantly higher than survivors (*P* < 0.001). An Ichikado CT score of more than 172 enabled prediction of mortality, with a sensitivity of 84.2% and a specificity of 79.7%. Multivariate analysis identified Ichikado CT score (HR, 7.772; 95% CI, 3.164–19.095; *P* < 0.001), together with age (HR, 1.030; 95% CI, 1.030–1.060; *P* = 0.043), as independent predictors of all-cause in-hospital mortality.

**Conclusions:**

Ichikado CT score is an independent predictor of both requiring invasive mechanical ventilation and all-cause mortality in patients hospitalized with COVID-19 pneumonia. Further prospective evaluation is necessary to confirm these findings.

*Trial registration:* The WCG institutional review board approved this retrospective study and patient consent was waived due to its non-interventional nature (Identifier: 20210799).

## Background

In late December 2019, a cluster of pneumonia cases of unknown origin was reported in Wuhan, Hubei Province, China [1]. Days later, the Chinese health authorities confirmed that coronavirus disease 2019 (COVID-19) was caused by severe acute respiratory coronavirus 2 (SARS-CoV-2) [2]. On March 11th 2020, the World Health Organization declared the COVID-19 outbreak a pandemic, as by then the cases of COVID-19 had spread to over 110 countries [3]. By February 14th 2021, the United States of America had a total of 27,221,607 cases of COVID-19, and 477,147 fatalities [4].

Coronavirus disease 2019 was initially thought to be a pulmonary-limited disease; however, we have learned that in moderate-to-severe cases it is accompanied by a systemic dysregulated inflammatory response and a hypercoagulable state [5–7]. The most common clinical presentation includes fever, cough, myalgias, fatigue and anosmia. While most patients will have mild symptoms and recover without medical management, some will develop dyspnea, requiring supplemental oxygen therapy, and eventually worsen to respiratory or multi-organ failure, requiring mechanical ventilation and intensive care unit (ICU) monitoring [8, 9]. As the number of people infected by SARS-CoV-2 continues to climb, early recognition of patients at risk of deterioration may aid in the triage and medical management of COVID-19 patients [10].

Most studies have focused on inflammatory biomarkers as predictive tools [11–13]. Data regarding prognostic value of chest computed tomography (CT) is scarce. To our knowledge, none of the chest CT scoring systems proposed for COVID-19 patients are quantitative and consider both the percentage of lung parenchyma affected and the type of infiltrate [10, 14–19]. In 2006, Ichikado et al. proposed and validated a chest CT scoring system for acute respiratory distress syndrome (ARDS) patients which proved to be an independent predictive factor for survival, ventilator-free days, and barotrauma incidence in mechanically ventilated patients [20].

The aim of this study was to determine the prognostic value of the Ichikado CT score upon admission for all-cause mortality in patients admitted to a COVID-19 ICU or intermediate care unit (IMU). We hypothesized that the Ichikado CT score is a useful predictive tool for hospital mortality.

## Methods

### Study population and design

We performed a single-center retrospective cohort study of patients with COVID-19 pneumonia admitted between March 20^th^, 2020 and October 31^st^, 2020. Two hundred forty-one patients with confirmed SARS-CoV-2 infection were admitted at our institution’s COVID-19 ICU and IMU. Infection was confirmed by reverse transcription polymerase chain reaction (RT-PCR), SARS Antigen Fluorescent Immunoassay (SOFIA), or IgG/IgM rapid tests, plus clinical and imaging correlation.

Patients were enrolled in our analysis if within 24 h from admission they underwent a chest CT, in supine or prone position, with or without intravenous contrast, and if within the same timeframe they had an arterial blood gas (ABG), a complete blood count, and a basic metabolic panel. Patients were excluded if they were younger than 18 years of age, they had a history of chronic interstitial lung disease or had missing demographic or outcome data. Considering the total patient population at our institution’s COVID-19 units, we calculated requiring a sample size of 149 patients to achieve a 95% confidence level.

The WCG institutional review board approved this retrospective study and patient consent was waived due to its non-interventional nature (Identifier: 20210799). This study was conducted in accordance with the amended Declaration of Helsinki.

### Data collection

Data were abstracted from both physical and digital hospital archives between February 24th, 2021 and February 25th, 2021. Two authors performed data entry to an electronic spreadsheet where information was double-checked. The demographic data included was age, gender, and race (African-American, Caucasian, Hispanic, Other). The clinical data included comorbidities like hypertension, diabetes mellitus, chronic obstructive pulmonary disease (COPD), chronic kidney disease (CKD) or end-stage renal disease (ESRD), the days since symptom onset until hospital admission, arterial partial pressure of oxygen (PaO_2_), arterial partial pressure of carbon dioxide (PaCO_2_), fraction of inspired oxygen (FiO_2_) at the time of ABG, arterial partial pressure of oxygen-to-fraction of inspired oxygen ratio (PaO_2_:FiO_2_), alveolar–arterial (A–a) gradient, Sequential Organ Failure Assessment (SOFA) score, absolute neutrophil count, absolute lymphocyte count, absolute neutrophil-to-lymphocyte ratio (NLR), Ichikado CT score, oxygen delivery device used at the time of CT scan and bloodwork obtention, medications utilized during hospitalization (corticosteroids, ascorbic acid, thiamine, anticoagulation, tocilizumab or remdesivir), prone positioning, renal replacement therapy (RRT), vasopressor use, acute kidney injury (AKI) during hospitalization, hospital length of stay (LoS) and new requirements for invasive mechanical ventilation. Fraction of inspired oxygen was estimated according to the oxygen delivery device used at the time of ABG. If patients had multiple laboratory measurements within the 24 h time frame, the worst clinical value was considered for this study.

Patients who underwent mechanical ventilation, before or after CT scan obtention, followed the low PEEP/high FiO_2_ ARDSnet protocol for lung protective ventilation, with additional attention to maintain a plateau pressure below 27 cmH_2_O and a driving pressure below 15 cmH_2_O [21]. Prone positioning was done in both intubated and non-intubated patients, following the institutional protocol of 16 h of prone position and 8 h of supine position.

### CT scan examination

Two independent radiologists with more than 10 years of experience of chest CT scan interpretation, who were blinded to clinical data, evaluated the chest CT scans included in our analysis. The observers followed a previously described qualitative and quantitative CT scoring system [20]. The visual scoring focused on the presence and extent of areas with normal lung parenchyma attenuation, ground-glass opacity (GGO), airspace consolidation, traction bronchiectasis or bronchiolectasis, and crazy-paving. Ground-glass attenuation was considered present if there was an area of opacification without obscuring the pulmonary vascular markings; airspace consolidation was defined as opacifications obscuring underlying vascular markings; traction bronchiectasis or bronchiolectasis were recognized as areas of irregularly dilated bronchi or bronchioles, respectively; presence of cystic air spaces with well-defined walls were defined as crazy-paving.

**Fig. 1:**
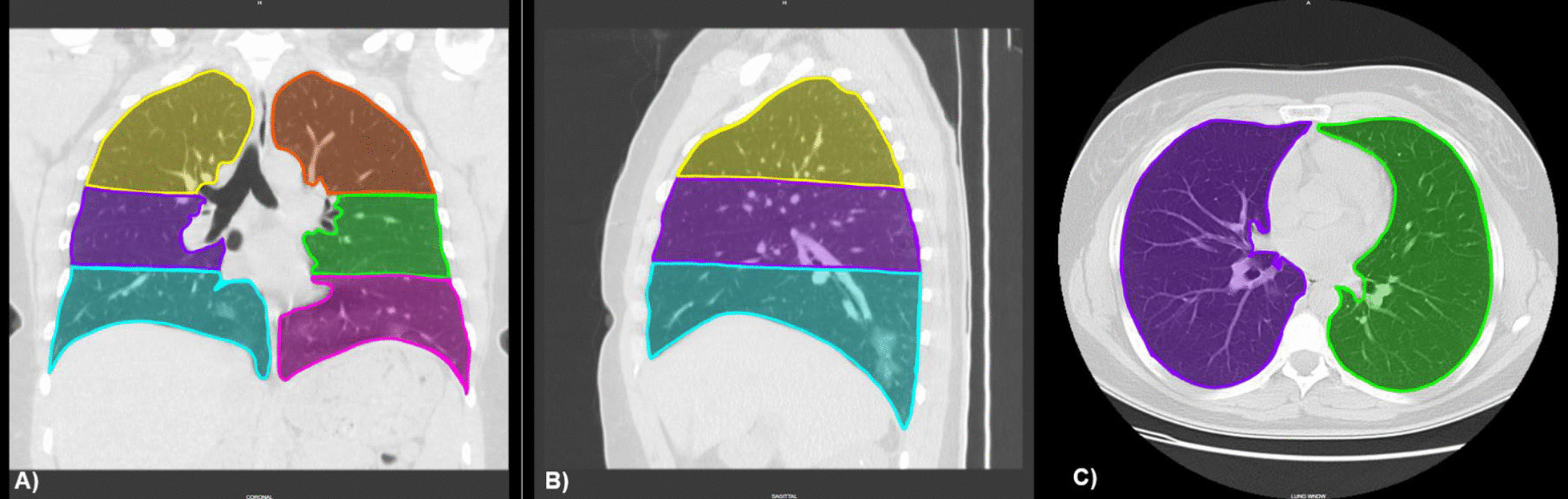
Ichikado pulmonary zones. The upper zone is defined as the lung parenchyma above the carina, the middle zone as the area between the carina and the pulmonary vein, and the lower zone as the infrapulmonary vein area. **A** Coronal lung window of CT chest showing all 6 Ichikado pulmonary zones. **B** Sagittal lung window of CT chest showing right lung Ichikado areas. **C** Axial lung window of CT chest showing right and left middle zones. This example shows images of a 34-year-old lady with coronavirus disease 2019

**Fig. 2:**
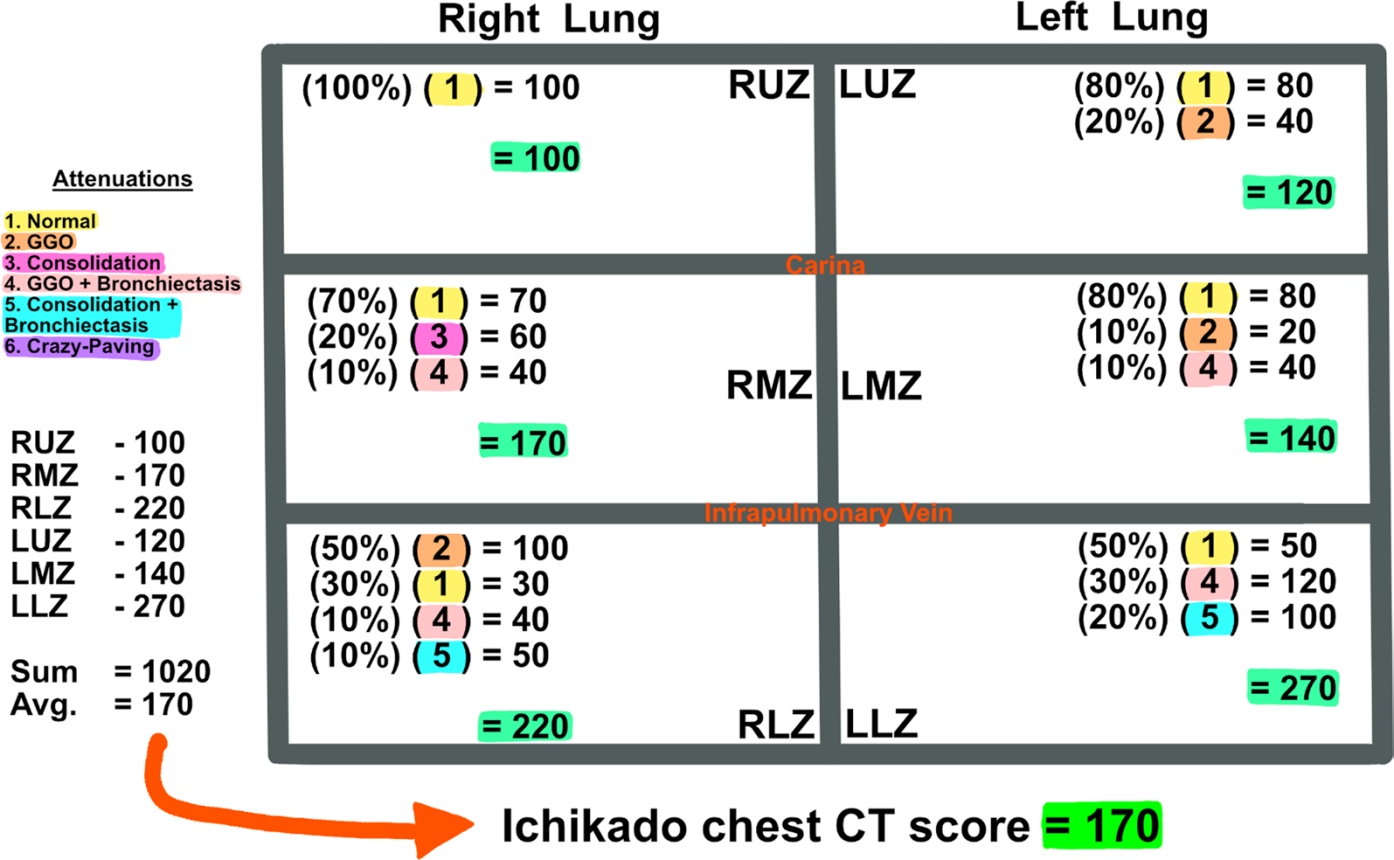
Example of Ichikado chest CT score calculation. *CT* Computed tomography, *RUZ* right upper zone, *RMZ* right middle zone, *RLZ* right lower zone, *LUZ* left upper zone, *LMZ* left middle zone, *LLZ* left lower zone, *GGO* ground-glass opacification

The previously described findings were graded on a scale of 1–6: 1, normal attenuation; 2, GGO; 3, airspace consolidation; 4, GGO with traction bronchiectasis or bronchiolectasis; 5, airspace consolidation with traction bronchiectasis or bronchiolectasis; 6, crazy-paving. Each attenuation was assessed in three (upper, middle, and lower) zones of each lung. The upper zone was defined as the lung parenchyma above the carina, the middle zone as the area between the carina and the pulmonary vein, and the lower zone as the infrapulmonary vein area (Fig. 1). The extent of each aberration was visually estimated to the nearest 10% of the lung parenchyma affected in each zone. The score for each zone was calculated by multiplying the percentage area by its respective point value (the score of 1 to 6). The six zones scores were averaged to determine an overall CT score for each patient (Fig. 2). The visual scoring of both radiologists was averaged, yielding a final score which was utilized in our analysis.

### Outcomes

The primary endpoint was all-cause mortality during hospitalization or survival to hospital discharge. Additionally, secondary endpoints were duration of hospital LoS, and new requirements for invasive mechanical ventilation after the time of CT examination.

### Statistical analysis

Continuous variables were expressed as mean ± standard deviation (SD) or median with interquartile range (IQR), depending on normality of distribution, and analyzed using a non-paired Student’s *t* test or Mann–Whitney U test, as appropriate. Categorical variables were expressed as frequencies and percentages, and compared using a Chi-squared test. Interobserver variability among radiologists, in chest CT scoring, was assessed with intraclass correlation coefficient. Correlations between Ichikado CT score and clinical data were determined using Spearman’s rank correlation coefficient.

Univariable and multivariable Cox regression models were used to test the association between potential independent predictive factors and all-cause mortality during COVID-19 hospitalization. Variables included in multivariable approach were those that have been reported with higher risk of adverse events; to avoid overfitting, we excluded factors for which *P* > 0.1 in univariable analysis, as well as those that significantly correlated with Ichikado CT score in Spearman’s analysis.

The Kaplan–Meier method was utilized to evaluate the relationship between CT score and all-cause mortality. Receiver operator characteristic (ROC) curve analysis was used to determine the optimal cutoff value of Ichikado CT score that yielded the highest sensitivity and specificity. The area under the ROC curve (AUC) was used to assess the performance of the discrimination models based on Ichikado CT score.

Statistical analysis was performed based on non-missing data using IBM SPSS, version 26.0, and missing data were not imputed. All tests were two-sided and a *P*-value < 0.05 was considered statistically significant. The authors assume responsibility for the accuracy and completeness of the data and analysis, as well as the fidelity of the study.

## Results

**Fig. 3:**
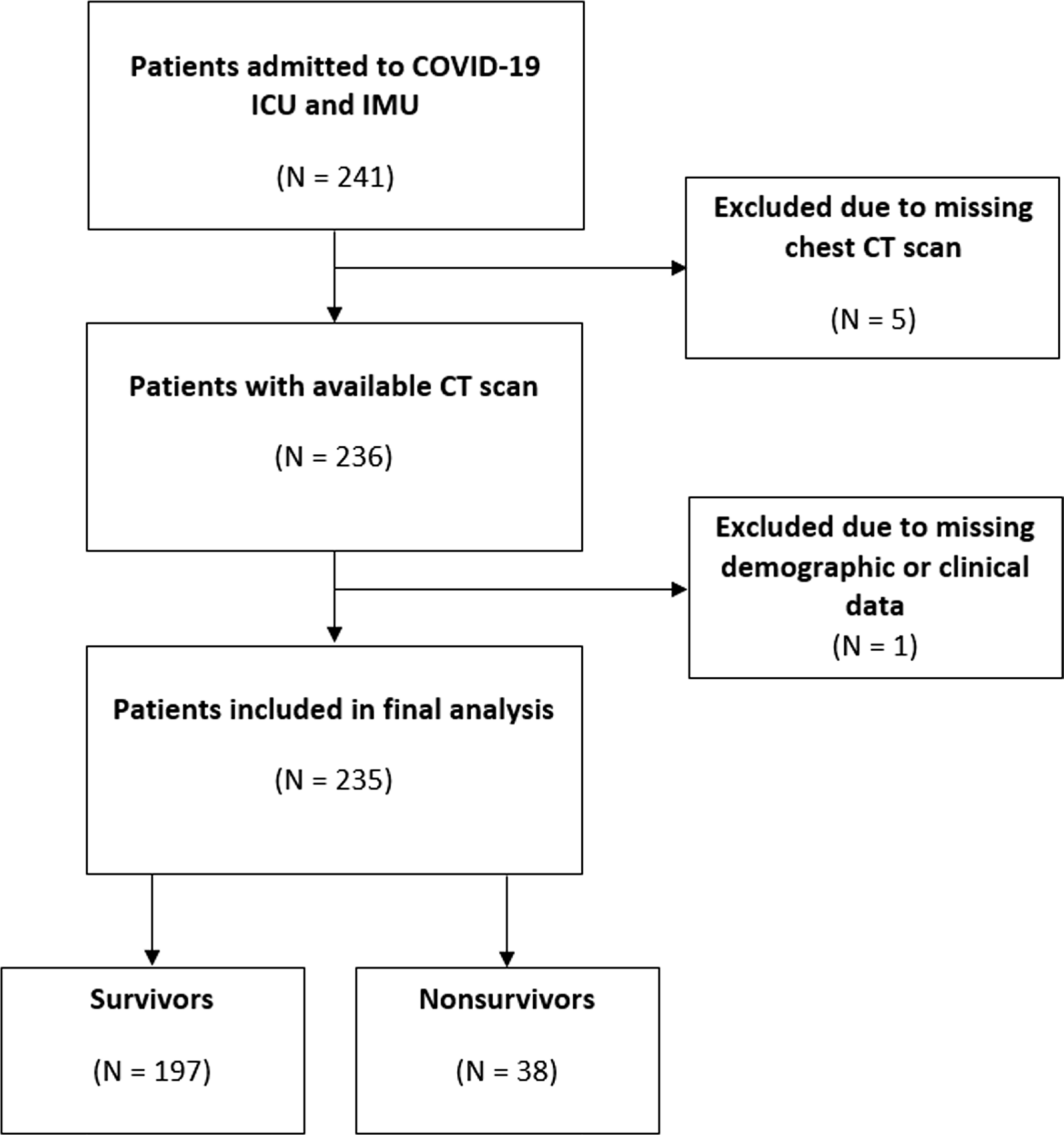
Flowchart of patient inclusion process

A total of 241 patients were admitted at our institution’s COVID-19 units, of which 235 were included in our analysis. Five patients were excluded due to missing chest CT scan within 24 h of admission, and one patient due to missing demographic or clinical data; none of the patients excluded died during hospitalization (Fig. 3). Demographic characteristics and clinical variables on the first 24 h from hospitalization for all 235 patients are reported in Table 1.

**Table 1:** Demographic and clinical characteristics of patients

Characteristic	All (*N* = 235)	Survivors (*N* = 197)	Nonsurvivors (*N* = 38)	*P*-value
Age (y)^†^	56.0 ± 15.6	53.9 ± 15.2	67.1 ± 13.1	**0.000**
Gender				**0.011**
Male^▼^	129 (54.9%)	101 (51.3%)	28 (73.7%)	
Female^▼^	106 (45.1%)	96 (48.7%)	10 (26.3%)	
Race				0.804
African-American^▼^	73 (31.1%)	60 (30.5%)	13 (34.2%)	
Caucasian^▼^	43 (18.3%)	38 (19.3%)	5 (13.1%)	
Hispanic^▼^	104 (44.2%)	86 (43.6%)	18 (47.4%)	
Other^▼^	15 (6.4%)	13 (6.6%)	2 (5.3%)	
Comorbidities				
Hypertension^▼^	119 (50.3%)	85 (43.1%)	34 (89.5%)	**0.000**
Diabetes mellitus^▼^	89 (37.9%)	62 (31.5%)	27 (71.1%)	**0.000**
COPD^▼^	80 (34.0%)	56 (28.4%)	24 (63.2%)	**0.000**
CKD^▼^	63 (26.8%)	38 (19.3%)	25 (65.8%)	**0.000**
ESRD^▼^	14 (6.0%)	11 (5.6%)	3 (7.9%)	0.582
PaO_2_ (Torr) ^‡^	76.0 (64.0–88.0)	77.0 (65.0–88.0)	70.5 (62.2–86.0)	0.216
PaCo_2_ (Torr) ^‡^	38.0 (35.0–41.0)	38.0 (35.0–41.0)	35.5 (33.0–40.2)	0.131
FiO_2_ (%) ^‡^	33.0 (28.0–40.0)	32.0 (28.0–40.0)	50.0 (40.0–100.0)	**0.000**
PaO_2_:FiO_2_^‡^	217.8 (157.5–275.0)	230.3 (171.1–285.7)	140.1 (70.2–215.6)	**0.000**
Mild (≥ 200)^▼^	132 (56.2%)	121 (61.4%)	11 (29.0%)	
Moderate (100–200)^▼^	67 (28.5%)	57 (28.9%)	10 (26.3%)	
Severe (< 100)^▼^	36 (15.3%)	19 (9.7%)	17 (44.7%)	
Oxygen delivery device				**0.000**
Room air^▼^	19 (8.1%)	19 (9.6%)	0 (0%)	
Nasal cannula^▼^	110 (46.8%)	101 (51.3%)	9 (23.7%)	
HFNC^▼^	81 (34.5%)	62 (31.5%)	19 (50%)	
BiPAP/CPAP^▼^	13 (5.5%)	9 (4.6%)	4 (10.5%)	
Non-rebreather mask^▼^	4 (1.7%)	3 (1.5%)	1 (2.6%)	
Endotracheal tube^▼^	8 (3.4%)	3 (1.5%)	5 (13.2%)	
Onset of symptoms to hospitalization (days)^‡^	7.0 (5.0–10.0)	7.0 (5.0–10.0)	10.0 (6.5–14.0)	**0.003**
Hospital length of stay (days)^‡^	10.0 (7.0–15.0)	9.0 (6.5–14.0)	12.0 (7.0–19.0)	**0.045**
AKI during hospitalization^▼^	59 (25.1%)	27 (13.7%)	32 (84.2%)	**0.000**
Ichikado CT score^‡^	146.7 (121.7–188.3)	138.3 (119.2–165.8)	215.8 (179.6–240.0)	**0.000**
A–a gradient (Torr)^‡^	119.7 (81.1–181.2)	109.3 (73.6–161.1)	235.7 (134.3–562.6)	**0.000**
SOFA score^‡^	3.0 (2.0–4.0)	3.0 (2.0–4.0)	4.0 (3.0–5.0)	**0.000**
Absolute neutrophil count × 10^9^/L^‡^	4.8 (3.4–7.4)	4.7 (3.3–6.5)	6.7 (3.6–10.7)	**0.005**
Absolute lymphocyte count × 10^9^/L^‡^	1.1 (0.7–1.7)	1.2 (0.8–1.7)	0.7 (0.6–1.0)	**0.000**
NLR^‡^	4.3 (2.7–8.3)	3.7 (2.5–6.9)	9.4 (5.9–13.3)	**0.000**
Treatment modalities				
Corticosteroids^▼^	235 (100%)	197 (100%)	38 (100%)	**–**
Ascorbic acid^▼^	235 (100%)	197 (100%)	38 (100%)	**–**
Thiamine^▼^	235 (100%)	189 (95.9%)	38 (100%)	**–**
Anticoagulation^▼^	227 (96.6%)	7 (3.6%)	38 (100%)	0.206
Tocilizumab^▼^	17 (7.2%)	7 (3.6%)	10 (26.3%)	**0.000**
Remdesivir^▼^	10 (4.3%)	197 (100%)	3 (7.9%)	0.225
Prone positioning^▼^	142 (60.4%)	109 (55.3%)	33 (86.8%)	**0.000**
RRT^▼^	31 (13.2%)	24 (12.2%)	7 (18.4%)	0.298
Vasopressor use^▼^	53 (22.6%)	17 (8.6%)	36 (94.7%)	**0.000**

For continuous variables, comparison of groups (survivors and nonsurvivors) was determined using the Student’s *t* test or Mann–Whitney U test, as appropriate. Chi-squared test was utilized for categorical variables. Statistically significant values (*P* < 0.05) are in bold. *A–a gradient* alveolar–arterial gradient, *NLR* absolute neutrophil-to-lymphocyte ratio, *AKI* acute kidney injury, *PaCO*_*2*_ arterial partial pressure of carbon dioxide, *PaO*_*2*_ arterial partial pressure of oxygen, *PaO*_*2*_*:FiO*_*2*_ arterial partial pressure of oxygen-to-fraction of inspired oxygen ratio, CPAP* BiPAP* bilevel positive airway pressure, *CKD* chronic kidney disease, *COPD* chronic obstructive pulmonary disease, continuous positive airway pressure, *ESRD* end-stage renal disease, *fio*_*2*_ fraction of inspired oxygen, *HFNC* high-flow nasal cannula, *RRT* renal replacement therapy, *SOFA score* Sequential Organ Failure Assessment Score

^**†**^Data are mean ± standard deviation

^**‡**^Data are median (interquartile range)

^**▼**^Data are frequency (%)

One hundred twenty-nine (54.9%) patients were male and 106 (45.1%) were female, with a mean age of 56.0 ± 15.6. The most common races were Hispanics (44.2%) and African Americans (31.1%), followed by Caucasians (18.3%). Hypertension (50.3%) and diabetes mellitus (37.9%) were the most common comorbidities, followed by COPD (34.0%), CKD (26.8%) and ESRD (6.0%). At the time of CT scan and bloodwork obtention, 110 (46.8%) patients were on nasal cannula, 81 (34.5%) on high-flow nasal cannula (HFNC), and 8 (3.4%) were endotracheally intubated. During hospitalization, 59 (25.1%) developed AKI. Forty-two (17.9%) patients had new requirements for invasive mechanical ventilation. One hundred ninety-seven (83.8%) patients survived to hospital discharge, while 38 (16.2%) died during their hospital stay. When compared to nonsurvivors, survivors were younger (*P* < 0.001), more likely to be women (*P* = 0.011) and had less days since onset of symptoms until hospitalization (*P* = 0.003). The median Ichikado CT score for nonsurvivors was 215.8 (179.6–240.0), which was significantly higher compared to survivors who had a median score of 138.3 (119.2–165.8) (*P* < 0.001). The interobserver variability among radiologists in calculating Ichikado CT score was excellent with an intraclass correlation coefficient of 0.992 (95% CI, 0.989–0.994; *P* < 0.001).

Analysis by Spearman’s correlation coefficient (*R*) indicated that Ichikado CT score was significantly associated with age (*R* = 0.243; *P* < 0.001), days from onset of symptoms to hospitalization (*R* = 0.270; *P* < 0.001), hospital length of stay (*R* = 0.414; *P* < 0.001), FiO_2_ (*R* = 0.425; *P* < 0.001), PaO_2_:FiO_2_ (*R* = -0.530; *P* < 0.001), A-a gradient (*R* = 0.481; *P* < 0.001), SOFA score (*R* = 0.260; *P* < 0.001), absolute neutrophil count (*R* = 0.361; *P* < 0.001), absolute lymphocyte count (*R* = -0.366; *P* < 0.001) and NLR (*R* = 0.493; *P* < 0.001).

**Fig. 4:**
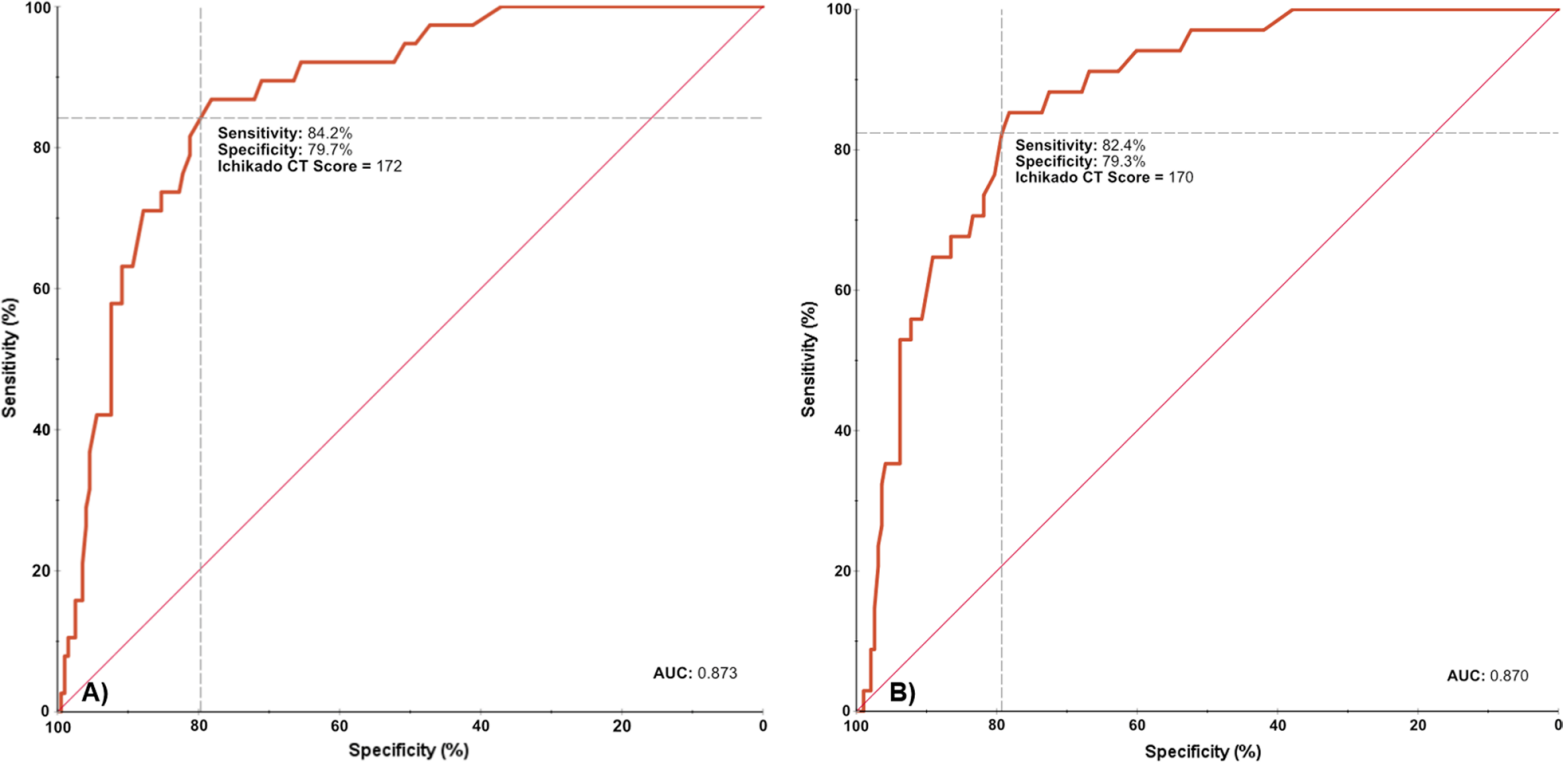
Receiver operator characteristic curves for cutoff values of CT scores with highest sensitivities and specificities. **A** Ichikado CT score of 172 has an 84.2% sensitivity and 79.7% specificity for predicting all-cause mortality; area under the curve (AUC) = 0.873. **B** Ichikado CT score of 170 has an 82.4% sensitivity and 79.3% specificity for predicting new requirement of invasive mechanical ventilation; AUC = 0.870

**Fig. 5:**
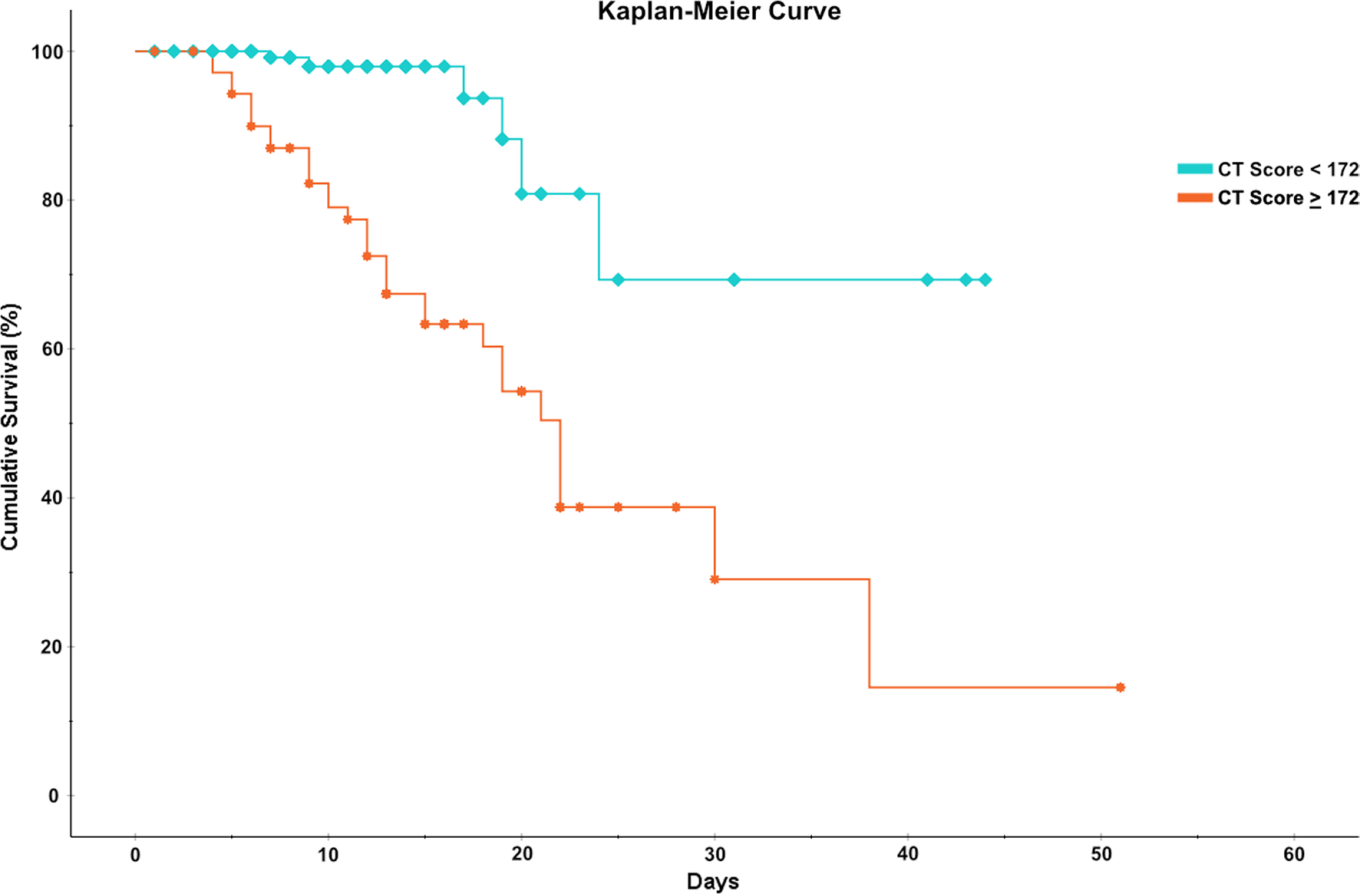
Kaplan–Meier survival curve. Cumulative survival rate comparison between COVID-19 patients with admission Ichikado CT score ≥ 172 and < 172. Percentage of cumulative survival is expressed on the *y*-axis, while time (days) of the observation period is expressed on the *x*-axis. *CT* Computed tomography

Receiver operator characteristic curve analysis yielded an optimal cutoff value of an Ichikado CT score of 172 for prediction of mortality, with a sensitivity of 84.2% and a specificity of 79.7% (AUC = 0.873); optimal cutoff value of CT score for prediction of new requirement of invasive mechanical ventilation was 170, with a sensitivity of 82.4% and a specificity of 79.3% (AUC = 0.870) (Fig. 4). A Kaplan–Meier survival curve was performed utilizing the ROC curve cutoff value for Ichikado CT score. By this method, a CT score ≥ 172 was predictive of all-cause hospital mortality (Fig. 5).

Table 2 summarizes univariable and multivariable Cox regression analysis results for all-cause mortality. On univariable analysis, an Ichikado CT score ≥ 172 was a statistically significant predictor for mortality with a hazard ratio (HR) of 6.864 (95% CI, 2.843–16.572; *P* < 0.001). Other significant variables for prediction of mortality were age (HR, 1.033; 95% CI, 1.010–1.058; *P* = 0.005), hypertension (HR, 4.621; 95% CI, 1.627–13.121; *P* = 0.004), diabetes mellitus (HR, 2.533; 95% CI, 1.248–5.139; *P* = 0.010), COPD (HR, 2.205; 95% CI, 1.135–4.285; *P* = 0.020), CKD (HR, 3.348; 95% CI, 1.698–6.602; *P* < 0.001), days from onset of symptoms to hospitalization (HR, 1.056; 95% CI, 1.005–1.109; *P* = 0.032), AKI during hospitalization (HR, 9.169; 95% CI, 3.806–22.085; *P* < 0.001), A-a gradient (HR, 1.003; 95% CI, 1.001–1.004; *P* < 0.001), SOFA score (HR, 1.189; 95% CI, 1.054–1.341; *P* = 0.005), absolute neutrophil count (HR, 1.123; 95% CI, 1.060–1.190; *P* < 0.001), absolute lymphocyte count (HR, 0.442; 95% CI, 0.220–0.891; *P* = 0.022), NLR (HR, 1.039; 95% CI, 1.021–1.058; *P* < 0.001), vasopressor use during hospitalization (HR, 33.935; 95% CI, 8.113–141.942; *P* < 0.001).

**Table 2:** Univariable and multivariable Cox regression analysis to predict all-cause mortality

	Univariable analysis			Multivariable analysis		
Variable	Hazard ratio	95% confidence interval	*P*-value	Hazard ratio	95% confidence interval	*P*-value
Age (y)	1.033	1.010–1.058	0.005	1.030	1.001–1.060	0.043
Gender						
Male	1.425	0.682–2.979	0.346	–	–	–
Female	1 (ref)					
Race			0.862	–	–	–
African-American	1 (ref)					
Caucasian	0.652	0.232–1.832	0.417			
Hispanic	0.808	0.394–1.654	0.559			
Other	0.916	0.206–4.072	0.908	–	–	–
Comorbidities						
Hypertension	4.621	1.627–13.121	0.004	2.056	0.665–6.359	0.211
Diabetes mellitus	2.533	1.248–5.139	0.010	2.004	0.940–4.273	0.072
COPD	2.205	1.135–4.285	0.020	–	–	–
CKD	3.348	1.698–6.602	0.000	–	–	–
ESRD	0.788	0.241–2.574	0.693	–	–	–
PaO_2_ (Torr)	0.991	0.975–1.007	0.284	–	–	–
PaCO_2_ (Torr)	1.005	0.971–1.040	0.789	–	–	–
FiO_2_ (%)	1.020	1.009–1.031	0.000	–	–	–
PaO_2_:FiO_2_	0.993	0.988–0.997	0.001	–	–	–
Oxygen delivery device			0.085	–	–	–
Room air	–	–	–			
Nasal cannula	0.194	0.064–0.587	0.004			
HFNC	0.459	0.169–1.242	0.125			
BiPaP/CPAP	0.383	0.102–1.441	0.156			
Non-rebreather mask	0.882	0.101–7.730	0.910			
Endotracheal tube	1 (ref)					
Onset of symptoms to hospitalization (days)	1.056	1.005–1.109	0.032	–	–	–
AKI during hospitalization	9.169	3.806–22.085	0.000	–	–	–
Ichikado CT score	1.010	1.005–1.014	0.000			
> 172	6.864	2.843–16.572	0.000		3.164–19.095	0.000
< 172	1 (ref)			7.772		
A–a gradient (Torr)	1.003	1.001–1.004	0.000	–	–	–
SOFA score	1.189	1.054–1.341	0.005	–	–	–
Absolute neutrophil count × 10^9^/L	1.123	1.060–1.190	0.000	–	–	–
Absolute lymphocyte count × 10^9^/L	0.442	0.220–0.891	0.022	–	–	–
NLR	1.039	1.021–1.058	0.000	–	–	–
Treatment modalities						
Steroids	–	–	–	–	–	–
Ascorbic acid	–	–	–	–	–	–
Thiamine	–	–	–	–	–	–
Anticoagulation	–	–	–	–	–	–
Tocilizumab	1.743	0.822–3.693	0.147	–	–	–
Remdesivir	1.162	0.354–3.811	0.805	–	–	–
Prone positioning	1.674	0.643–4.359	0.291	–	–	–
RRT	0.812	0.354–1.860	0.622	–	–	–
Vasopressor use	33.935	8.113–141.942	0.000	–	–	–

Multivariate analysis showed that Ichikado CT score (HR, 7.772; 95% CI, 3.164–19.095; *P* < 0.001), together with age (HR, 1.030; 95% CI, 1.030–1.060; *P* = 0.043), were independent predictors of all-cause in-hospital mortality. Additionally, an Ichikado CT score ≥ 172 (HR, 22.480; 95% CI, 8.161–61.921; *P* < 0.001) was an independent predictor of new requirement of invasive mechanical ventilation during hospitalization.

## Discussion

Ichikado et al. previously validated a CT score in patients with ARDS, identifying a score above 230 as an independent predictor of mortality [20]. In our single-center retrospective cohort study, we recognized the Ichikado CT score as an independent predictor of both requiring mechanical ventilation and all-cause mortality in patients hospitalized with COVID-19 pneumonia. However, our cutoff value with highest sensitivity and specificity for prediction of mortality was 172. We believe this difference is explained mainly by the time to obtention of the CT scans. In contrast with their study, which analyzed scans up to 7 days from the onset of ARDS, we only included CT scans taken in the first 24 h of hospital admission. It has previously been described that COVID-19 CT imaging features will vary drastically according to the stage of disease. Early on, patients will have predominantly small areas of GGOs, compared to the late phase of disease (12–17 days after onset of symptoms), during which patients will have bilateral consolidations mixed with GGOs, and even crazy-paving pattern [22, 23]. The presence of fibroproliferative attenuations in chest CT scans obtained in later stages of the disease, could potentially explain the discrepancies between their study and ours. In addition, the CT findings of “typical” ARDS differ considerably from that of COVID-19 organizing pneumonia [24].

Multiple CT scoring models have been proposed in COVID-19 for quantifying severity of disease, and predicting prognosis. Regardless of the CT scoring system analyzed, all prior studies have identified a CT score as an independent predictor of adverse outcome in patients with COVID-19 pneumonia [10, 15, 17, 18]. Moreover, CT scores had significant associations with various inflammatory biomarkers known to be predictors of mortality [14, 16]. However, to our knowledge, no other study has followed the Ichikado scoring method, which we believe might be superior to previously described methods due to its reproducibility and having both a quantitative and qualitative approach.

Our multivariate regression results showed that an Ichikado CT score ≥ 172 was the highest independent predictor of all-cause in-hospital mortality. Furthermore, a higher absolute lymphocyte count at the time of hospital admission was associated with significantly decreased odds of mortality. These findings are consistent with prior reports [25–27].

The role of chest CT scan in the diagnosis and management of COVID-19 remains controversial. While some authors and radiological societies recommend against routine use of CT scan in patients with COVID-19, our analysis seems to suggest a benefit by allowing early recognition of patients at high risk of decompensation [28, 29]. Additionally, the high diagnostic sensitivity may prove to be an asset as CT scans may show characteristic findings of the disease even when RT-PCR is negative (false-negative), or in hospitals with a relatively long turn-around time for RT-PCR results [16, 30].

There were limitations in our analysis. Due to the single-center retrospective observational nature of the study, we cannot exclude the possibility of unmeasured confounders. Furthermore, despite including laboratory data obtained on the same day as the chest CT, we did not provide correlation analysis with biomarkers (e.g., interleukin-6, C-reactive protein, D-dimer, ferritin, etc.) known to be predictors of mortality in COVID-19. External validation of our analysis is required as most of the studied patients, unless medically contraindicated, received the MATH + protocol (methylprednisolone, IV ascorbic acid, thiamine, heparin, etc.) which is not the standard of care in most institutions, and could potentially be a confounder in our study [31, 32].

## Conclusions

Further prospective evaluation of the Ichikado CT score in patients with COVID-19 will be necessary not only to clarify its prognostic value, but also to assess its potential application in stratifying severity of disease, guiding treatment, and monitoring disease progression. Ideally, such investigations would help identify the patients at high risk of deterioration who require prompt initiation of treatment and ICU monitoring.

In summary, this study provided evidence that Ichikado CT score obtained in the first 24 h of hospital admission, is an independent predictor of requiring invasive mechanical ventilation and all-cause in-hospital mortality in patients with COVID-19 pneumonia. Since COVID-19 is a potentially fatal disease, utilizing the Ichikado CT score may aid in appropriately triaging patients, so that those with severe disease can timely initiate more aggressive treatment under closer monitoring.

## Data Availability

The datasets used and analyzed during the current study are available from the corresponding author on reasonable request.

## Abbreviations

95% CI: 95% Confidence interval
A–a gradient: Alveolar–arterial gradient
ABG: Arterial blood gas
AKI: Acute kidney injury
ARDS: Acute respiratory distress syndrome
AUC: Area under roc curve
CKD: Chronic kidney disease
COPD: Chronic obstructive pulmonary disease
COVID-19: Coronavirus disease 2019
CT: Computed tomography
ESRD: End-stage renal disease
FiO_2_: Fraction of inspired oxygen
GGO: Ground-glass opacity
HFNC: High-flow nasal cannula
HR: Hazard ratio
ICU: Intensive care unit
IMU: Intermediate care unit
IQR: Interquartile range
LoS: Length of stay
MATH + protocol: Methylprednisolone, IV ascorbic acid, thiamine, heparin, etc.
NLR: Absolute neutrophil-to-lymphocyte ratio
PaCO_2_: Partial pressure of arterial carbon dioxide
PaO_2_: Partial pressure of arterial oxygen
PaO_2_FiO_2_: Arterial partial pressure of oxygen-to-fraction of inspired oxygen ratio
*R*: Spearman’s correlation coefficient
RRT: Renal replacement therapy
ROC curve: Receiver operator characteristic curve
RT-PCR: Reverse transcription polymerase chain reaction
SARS-CoV-2: Severe acute respiratory syndrome coronavirus 2
SD: Standard deviation
SOFA Score: Sequential Organ Failure Assessment Score
SOFIA: SARS antigen fluorescent immunoassay
